# Food Safety—Transcriptomics and Proteomics

**DOI:** 10.3390/ijms242417127

**Published:** 2023-12-05

**Authors:** Mónica Carrera

**Affiliations:** Food Technology Department, Institute of Marine Research (IIM), Spanish National Research Council (CSIC), 36208 Vigo, Pontevedra, Spain; mcarrera@iim.csic.es; Tel.: +34-(98)-6231930

Food safety is a critical aspect of public health and involves the handling, preparation, and storage of food to avoid contamination and foodborne illnesses [1]. From farm to fork, the journey of food products involves several stages, each of which is crucial for ensuring food safety [2]. Foodborne diseases caused by bacteria, parasites, and viruses continue to be a significant concern worldwide. These diseases can result from the consumption of contaminated food and water, and they pose a considerable burden for public health. Common pathogens associated with foodborne illnesses include *Salmonella*, *Escherichia coli* (*E. coli*), *Campylobacter*, *Listeria*, norovirus, and various parasites [3]. In addition, the incidence of food allergies has indeed been increasing over the last 20 years, impacting a significant number of individuals globally [4]. Preventing foodborne illnesses and managing food allergies involve a combination of regulatory measures, research institutions, good industrial and manufacturing practices, proper food handling and preparation at home, and public awareness [5]. Government agencies, food producers, researchers, and consumers all play crucial roles in minimizing the risks associated with foodborne diseases and allergies [6].

Transcriptomics and proteomics are powerful molecular biology techniques that have shown great promise in advancing our understanding of various biological processes, including those related to food safety [7]. These methodologies have contributed significantly to the identification of potential hazards, the assessment of food quality, and the development of strategies to enhance food safety [8]. Transcriptomics involves the analysis of the whole set of RNA transcripts in a cell or organism. It provides insights into gene expression patterns, allowing researchers to understand how genes are activated or suppressed in response to different conditions, including foodborne pathogens [9]. Proteomics emphasizes the identification and quantification of the global set of proteins in a specific biological system [10]. It offers a comprehensive view of the functional proteins present, which is crucial for understanding various biological processes. These methodologies permit the detection of food parasites [11], food authenticity investigation [12], food microorganism detection [13], recognition of food allergens [14], food processing analysis [15], by-product safety [16] and systems biology studies [17], among others [18]. Thus, these approaches are a beneficial proposal for food science findings, where agencies, investigation laboratories, food companies, and controlling institutions are merging efforts to obtain essential understandings about food safety. The prospectives of transcriptomics and proteomics in food safety are emphasized in this Special Issue on several topics regarding food safety. The six research articles that are included in this Special Issue, “Food Safety—Transcriptomics and Proteomics”, offer a brilliant synopsis of the extensive range of transcriptomics and proteomics approaches employed to food safety.

In this perspective, the article reported by Chang et al., “Quantitative proteomic analysis on the slightly acidic electrolyzed water triggered viable but non-culturable *Listeria monocytogenes*”, reports the impact of slightly acidic electrolyzed water (SAEW), as an efficient disinfectant of the food industry, on *Listeria monocytogenes* [19]. *Listeria monocytogenes* is a species of bacteria that can cause a serious infection known as listeriosis [20]. *Listeria* is a Gram-positive, rod-shaped bacterium, and it is facultatively anaerobic, meaning it can grow in both the presence and absence of oxygen. Unlike many other bacteria, *Listeria monocytogenes* is capable of growing at refrigeration temperatures, which makes it a concern for food safety. The authors performed an interesting quantitative proteomics investigation using tandem mass tags (TMT) [21] in an Orbitrap Fusion Tribrid equipment to identify differentially regulated proteins under different conditions of SAEW. SAEW is a type of water that has undergone electrolysis to produce a solution with a slightly acidic pH. Electrolysis involves passing an electric current through a water solution, causing chemical reactions to occur at the electrodes. The resulting water can have different properties depending on the pH and the presence of ions. The acidity of SAEW can contribute to its antimicrobial properties, making it effective against certain bacteria and viruses. SAEW is commonly used to disinfect surfaces, equipment, and even food products due to the fact that it can help to reduce the presence of pathogens [22]. The functional enrichment investigation of the differentially regulated proteins presented in this article showed that ribosomes, aminoacyl-tRNA biosynthesis, and biosynthesis of secondary metabolites were enriched functions influenced by SAEW. Additionally, the authors investigated the task of protein chlorination, a possible concern of reactive chlorine species produced throughout the SAEW creation course, by recognizing 31 chlorinated peptides and 22 proteins that were functionally enriched in translation. The authors suggested that SAEW might induce modifications in the protein translation procedure and activate processes that counteract ribosome biosynthesis. For this, the authors also offer potential strategies of control of *Lysteria monocytogenesis* using SAEW.

The manuscript published by Huang et al., “Comparative anatomical and transcriptomics reveal the larger cell size as a major contributor to larger fruit size in apricot”, presents the comparison of transcriptomics changes of fruit growth and expansion in two apricot cultivars (small-fruit and large-fruit) [23]. After the transcriptomics examination, crucial differentially expressed genes most probable to effect cell size were selected, including genes immersed in cell wall loosening mechanisms and auxin signal transduction [24]. In the study, bigger cell size was recognized as the main factor responsible for larger fruit size in apricot, and some cell wall loosening-correlated genes were recognized as key applicants for managing cell size. Interestingly, the authors observed expression alteration in numerous genes described to be implicated in endo-reduplication between the two apricots, such as CDCs, APCs, CDKs, and CYCs. The authors conclude that the transcriptomics analysis of the expression ranks of genes implicated in fruit size allows new insight into the genetic enhancement and molecular evolutionary and breeding processes of apricot.

The article written by Abril et al., “Shotgun proteomics analysis, functional networks, and peptide biomarkers for seafood-originating biogenic-amine-producing bacteria”, reports the shotgun proteomics characterization of biogenic amine-producing bacteria from seafood for the first time [25]. Biogenic amines are organic bases that are created through the decarboxylation of amino acids [26]. Some bacteria have the capacity to generate biogenic amines as by-products of their metabolism. Biogenic amines can be found in various foods and can have implications for food safety, as some of them are associated with adverse health effects when consumed in high amounts [27]. Histamine, tyramine, putrescine, and cadaverine are examples of biogenic amines that can be produced by certain bacteria [28]. The existence of elevated levels of biogenic amines in certain foods can be a concern, as excessive intake of these amines has been linked to symptoms such as headaches, nausea, and allergic reactions in susceptible individuals. The authors used a shotgun proteomics method for the characterization of diverse biogenic-amine-producing bacteria from seafood. For this purpose, they utilized a liquid chromatography–electrospray ionization tandem mass spectrometry (LC–ESI–MS/MS)-based workflow using an LTQ-Orbitrap XL instrument to identify 10,673 peptide spectrum matches fitting to 4081 peptides and corresponding to 1811 proteins. Significant functional pathways were defined, and strains were differentiated into hierarchical groups. An estimated interactomics network was also generated using STRING (260 nodes/1973 interactions) [29]. Most of the designed proteins were related with networks/pathways of putrescine metabolism, energy, and host–virus interaction. In addition, 77 species-specific peptide biomarkers relating to 64 different proteins were suggested to detect 10 bacteria. The authors concluded that this article symbolizes the global proteomic dataset of biogenic-amine-producing strains presented in seafood.

In the article written by Pérez-Polo et al., “Identifying natural bioactive peptides from the common octopus (*Octopus vulgaris* Cuvier, 1797) skin mucus by-products using proteogenomic analysis”, the authors present an outline of the protein identification of skin mucus by-products of the common octopus using a shotgun proteogenomics approach [30]. The skin mucus of the common octopus (*Octopus vulgaris*) serves various purposes, including protection, communication, and camouflage [31]. Using an Orbitrap-Elite mass spectrometer, the authors identified a total of 510 proteins, merging from 5937 identified spectra of 2038 different peptides. The final proteome assembling was examined in integrated in silico studies, comprising KEGG pathway analysis, gene ontology (GO) using PANTHER [32], network studies using STRING [29], and prediction/characterization of potential bioactive peptides [33]. Bioactive peptides are small sequences of amino acids that can exert physiological effects in the body. They can have various functions, including antimicrobial activity, antioxidant properties, and involvement in cell signaling. The exploration of bioactive peptides in marine organisms, including cephalopods like octopuses, has gained attention due to their potential pharmaceutical and biotechnological applications. The results obtained in this work showed proteins closely correlated with defense and several potential bioactive peptides with antimicrobial properties. Antimicrobial peptides (AMPs) are a miscellaneous collection of molecules that play a relevant role in the innate immune system of various organisms, including marine animals [34]. These peptides exhibit antimicrobial activity against bacteria, fungi, and other microorganisms. Understanding the antimicrobial peptides present in the skin mucus can provide insights into the octopus’s defense mechanisms against pathogens in its environment.

The manuscript published by Quintela-Baluja et al., “Rapid proteomic characterization of bacteriocin-producing *Enterococcus faecium* strains from foodstuffs”, presents the characterization of two bacteriocin-producing *Enterococcus faecium* strains using a liquid chromatography instrument coupled to a trapped ion mobility spectrometry–time-of-flight mass spectrometry instrument (TimsTOF) [35]. *Enterococcus faecium* is a lactic acid bacteria (LAB) belonging to the genus *Enterococcus* [36]. LAB are a group of bacteria that generate lactic acid as a by-product of carbohydrate fermentation. LAB are widely used in various food fermentation processes and are also known for their probiotic properties [37]. In the present work, the authors identified more than 1100 unique proteins per isolate. Quantitative proteomics was also performed on bacteriocins [38], proteins providing resistance to antibiotics, heavy metals, and virulence factors. The characterization of these bacteria displayed great potential for defining and improving the bioengineering and biotechnology properties of other LAB strains in the food industry. The authors concluded that the present workflow could be converted into a gold standard for food safety, defining and improving the biotechnology and bioengineering properties of LAB in the food industry.

Finally, the article published by Abril et al., “Proteomic characterization of virulence factors and related proteins in *Enterococcus* strains from dairy and fermented food products”, presents the characterization of virulence factors of the pathogenic *Enterococcus* strains using shotgun proteomics [39]. Virulence factors are specific characteristics or molecules produced by pathogens that enable them to cause disease or infection in a host organism [40]. These factors enhance the pathogen’s ability to colonize, invade the host immune system, and cause damage to host tissues. Different pathogens possess distinct sets of virulence factors tailored to their specific strategies for survival and replication within a host. The current study illustrates the use of LC–ESI–MS/MS in an LTQ-Orbitrap XL instrument to perform a comprehensive shotgun proteomics representation for opportunistic pathogenic *Enterococcus* from diverse fermented food and dairy foodstuffs. A total of 310 peptides related to proteins completing a direct task as virulence factors for *Enterococcus* pathogenicity were identified. Understanding the specific virulence factors of a pathogen is crucial for developing effective strategies for prevention, diagnosis, and treatment of infectious diseases. Researchers often study these factors to identify targets for further vaccines or therapeutic interventions.

This Special Issue entitled “Food Safety—Transcriptomics and Proteomics” is a perfect and suitable compendium for researchers looking for to understand the transcriptome and proteome in the context of food safety of any food biological sample. In summary, this Special Issue is designed to be a valuable resource for researchers in the field of food safety who are interested in applying transcriptomics and proteomics approaches to analyze and understand the molecular aspects of various food samples. Researchers in this area can expect to find relevant articles and insights within this Special Issue to enhance their understanding of food safety from a molecular perspective. To conclude, the Guest Editor, Dr. Carrera, desires to express her appreciation to all the authors for their involvement in the creation of this Special Issue.
